# Effects of the Powder from Hoggery Desulfurization Tanks on the Salinity Resistance of Lettuce

**DOI:** 10.3390/plants11070868

**Published:** 2022-03-24

**Authors:** Yao-Tsung Chang, Yong-Hong Lin, Wei-Jia Wang

**Affiliations:** 1 Department of Crop Environment, Kaohsiung District Agricultural Research and Extension Station, Pingtung 908126, Taiwan; ytc@mail.kdais.gov.tw; 2 Department of Plant Industry, National Pingtung University of Science and Technology, Pingtung 912301, Taiwan; ga36221212@gmail.com

**Keywords:** agricultural management, circular economy, mineral nutrition, salinity, sustainable growth

## Abstract

Lettuce is an important vegetable cultivated worldwide, even in regions with highly saline soils. A large amount of research discusses the application of sulfur on the increase of antioxidation in plants. The powder from hoggery desulfurization tanks contained high amounts of sulfur and small amounts of other nutrients for plants. This powder can be added to liquid fertilizer to create high-sulfur liquid fertilizer (HSLF). This study observed the cell morphologies of lettuce root apices under salt stress after the application of HSLF. Lettuce plants were cultivated in hydroponic solutions containing one of two NaCl (0 and 40 mM) and three HSLF (0.0, 1.5, and 3.0 g L^−1^) concentrations. Salinity reduced the K^+^/Na^+^ ratio in the plant leaves; however, this reduction was smaller in the HSLF-treated plants. Except for phosphate and potassium, nutrient absorption is inhibited under conditions of high salinity. Using scanning electron microscopy, we observed apices more integrated on cell roots after increasing HSLF supplement under non-salt-stressed conditions. In addition, the cells were repaired after increasing the supplement of HSLF under the condition of 40 mM NaCl. Although salt stress reduced plant growth, the reductions were minimized in the HSLF-treated plants. The application of HSLF potentially alleviated salt injury in lettuce root apices and was probably associated with the improvement of phosphorus and potassium absorption and increasing K^+^/Na^+^ ratios in lettuce plants.

## 1. Introduction

Lettuce is an edible leaf vegetable that is extensively cultivated worldwide. Lettuce contains many nutrients crucial for human metabolism, such as potassium, calcium, iron, manganese, zinc, magnesium, and compounds with biological activity [1]. Soil salinity has gradually become a severe threat to global agriculture. Plants grown in saline soils must be able to achieve nutrient uptake under the high osmotic potential of the saline soil [2]. Lettuce has a moderate level of resistance to salinity [3]. Harvest production gradually decreases as the electrical conductivity of the soil increases. Soil salinity is highly detrimental to all aspects of plant growth [4]. Salt stress reduces the water potential of roots, and sodium causes physiological disorders and nutritional deficiency in plants [5]. Additionally, the lower levels of functional proteins in lettuce grown in high-salinity conditions result from the actions of reactive oxygen species [6]; such low protein levels affect both the photosynthesis and respiration of plants, which in turn influence growth.

Sulfur is a key element of plant physiology. For example, sulfur affects the metabolism of methionine, cysteine, vitamins, iron–sulfur agglomerates, sulfoxides, glucosinolates, and glutathione [7]. The foliar applications of fertilizers containing different forms of sulfur: elemental S^0^, sulphate SO_4_^2–^ and thiosulphate S_2_O_3_^2–^ (in combination with other macro- and microelements), and fungicides with (S^0^ + F) or without sulfur in the conventional fungicide program (F). The application of the conventional fungicide program (S^0^ + F and F) is more effective against scab incidence of apple trees than the inorganic forms of sulfur alone. When sulfur is supplied in an appropriate dose, the salt resistance of barley [8] and mustard [9,10] improves, with an increase in glutathione and the activity of antioxidant enzymes in the high-salinity environment compared with plants not treated with sulfur. Moreover, sulfur’s antioxidative effect that reduces salt damage may relate to the sufficient absorption of potassium and phosphorus and a high K^+^/Na^+^ ratio [11].

Biodesulfurization is a method of removing sulfur from the wastewater of the anaerobic fermentation process for transforming the methane from a hoggery into electricity [12]. Hydrogen sulfide corrodes the metal components of electric generators and causes the engine lubrication to deteriorate [13]. The hydrogen sulfide in biogas mainly originates from the biodegradation of sulfur-containing proteins or the desulfurization of sulfuric acid. The concentration of hydrogen sulfide in pig manure is approximately 2000–6000 ppm [14]. Therefore, before the transfer of biogas to an electrical power generator, the hydrogen sulfide must be removed. This sulfur-containing HSLF can be used in the production of fertilizer and materials for biological pest control [15]. The present study evaluated the complex mechanism of saline resistance in lettuce grown in a HSLF-containing liquid fertilizer.

## 2. Materials and Methods

### 2.1. Design of Experiment

The experiment was conducted in a growth chamber at the plant nutrition laboratory at the Department of Plant Industry at National Pingtung University of Science and Technology in Taiwan. Lettuce seeds (*Lactuca sativa* L.) were sown with deionized water obtained from Fonnong Co. (Pingtung, Taiwan). After 7 days, the seedlings were transferred to glass containers containing 5 L of Hoagland hydroponic solution [16]. The Hoaglang solution contains N = 98 mg/L, P = 93 mg/L, K = 117 mg/L, Ca = 42 mg/L, Mg = 24 mg/L, S = 65.6 mg/L, Fe = 1.1 mg/L, Mn = 0.27 mg/L, B = 0.27 mg/L, Cu = 0.03 mg/L, Zn = 0.13 mg/L. This sulfur content could almost be ignored when sulfur powder was added to Hoagland solution. After two weeks, the uniform seedlings were selected and transferred to glass containers (one seedling per container) containing 3 L of hydroponic solution. The experiment involved applying hydroponic solution with 0 (S_0_) or 40 (S_40_) mM NaCl concentrations and 0 (H_0_), 1.5 (H_1.5_), and 3.0 (H_3.0_) g L^−1^ HSLF concentrations derived from the powder from a hoggery. The actual doses of sulfur in the hydroponic solution and container were 1.28 g/L and 2.56 g/L, respectively. The treatments were denoted as S_0_H_0_, S_0_H_1.5_, S_0_H_3.0_, S_40_H_0_, S_40_H_1.5_, and S_40_H_3.0_. The HSLF contained Na = 3918 mg/L, NH_4_^+^ = 300.4 mg/L, K^+^ = 114.3 mg/L, Ca^+2^ = 545.1 mg/L, Mg^+2^ = 911.2 mg/L, Cl^−1^ = 132.1 mg/L, NO_2_^−1^ = 4.67 mg/L, Br^−1^ = 63.1 mg/L, NO_3_^−^ = 115.3 mg/L, PO_4_^−3^ = 2691.2 mg/L, Li^+1^ = 53.7 mg/L, S = 52,000 mg/L. The treatments were applied 2 weeks after the plants were transferred to the containers, and all treatments were performed in triplicate. The daytime temperature, nighttime temperature, and relative humidity of the air in the growth chamber were 28.5 °C ± 0.6 °C, 22.4 °C ± 1.3 °C, and 66.5%, respectively. The nutrient solutions were replaced every 4 days, and the daily volume lost through evapotranspiration was replaced by adding distilled water. The pH of the hydroponic solutions was maintained at approximately 5.6–5.8 and adjusted with a 0.5 M sodium hydroxide solution daily. The experiment proceeded for three weeks.

### 2.2. Mineral Content

Leaf samples were digested using a biomass digester at 350 °C. First, 0.2 g of dry, ground leaf powder was added to digestion tubes with 4.0 mL of 98% sulfuric acid [17]. The nitrogen (N) was determined by Kjeldahl method. The K and Na contents were determined using an inductively coupled plasma–optical emission spectroscopy instrument (JY-2000). Phosphorus content was determined through the blue molybdenum method at an absorbance wavelength of 660 nm, based on a standard curve adjusted with solutions of increasing KH_2_PO_4_ concentration.

### 2.3. Cell Morphology of Root Apices in Lettuces

The root apices (1 cm) were cut from the lettuce grown under different treatments, and the cell morphology was observed using scanning electron microscopy (SEM). The procedure was as follows: Sodium phosphate buffer (0.1 M) containing 2.5% glutaraldehyde (pH 7.0) was prepared for fixation of the lettuce root apices for 2 h. Subsequently, a prepared mixture of 0.1 M sodium phosphate buffer and 5% sucrose at pH 7.0 was used to rinse the root apices for 10 min. Before SEM, the samples were fixed for 1 h with osmium tetroxide (1%) containing 0.1 M sodium phosphate at pH 7.0. The samples were dehydrated with deionized water, and then 50–100% alcohol and 100% acetone were added. Finally, the samples were embedded in Spurr’s resin before being cut with an ultramicrotome (Reichert-Jung Ultracut E) and dyed using lead citrate-uranyl acetate. The phosphorus and potassium on the root surface were analyzed through energy-dispersive spectroscopy (EDS). EDS analysis is based on the intersection of X-rays with samples. Because the atomic structures of various elements differ, the spectra of those elements can be distinguished, and the content of such elements in samples can be determined.

### 2.4. Statistical Analysis

A completely randomized design was used for the experiment, with three replicates. The variables were the 0 and 40 mM NaCl concentrations and 0.0, 1.5, and 3.0 g L^−1^ HSLF concentrations. Statistical analysis was performed using the SAS package. The mean values (*p* < 0.05) were used for Duncan’s multiple-range test.

## 3. Results

### 3.1. Growth Analysis

Table 1 lists the root length, plant height, and dry weight of the lettuce plant. However, significant differences were observed across salt and HSLF treatments (*p* < 0.05). In the treatment of non-salt-stressed plants, the supplement of 3.0 g L^−1^ HSLF increased height by approximately 5.1%, root length by 3.3%, and fresh weight by 21.6% more than the treatment of no HSLF. However, these increases were not significant. Compared with the control plants (S_0_F_0_), the application of salt stress yielded significantly lower plant height, root length, and fresh weight by 10.1%, 5.0%, and 14.0%, respectively (Table 1). However, the supplement of 3.0 g L^−1^ HSLF attenuated this negative effect on plant growth. By contrast, root growth was influenced only by HSLF fertilization (*p* < 0.05), without significant association with the other factors. Plants fertilized with HSLF at 3.0 g L^−1^ had the greatest fresh root weight of the six samples, with a mean 21.6% higher than that of the nonsupplemented plants. Phosphorous content in the non-saline treatment was higher than that of the saline treatment. Potassium content in high-saline treatment was lowest; however, the content increased significantly after the addition of HSLF. The non-salt-stressed plants did not exhibit significant differences in nutrient content; however, the salt stress yielded slightly lower phosphorus and potassium content, a phenomenon that was reversed through HSLF supplementation.

### 3.2. Mineral Content in Lettuce

Table 2 shows that the Na^+^ content of roots and leaves in the non-salt-stressed lettuce fertilized with HSLF was slightly and not significantly higher than that in the control plants; however, the mean values were significantly higher in the plants treated with NaCl (40 mM). The Na^+^ content in the salt-stressed plant leaves was lower with greater concentrations of HSLF. Furthermore, the salt-stressed leaves had lower K^+^ content than the control plants; however, that value was significantly higher in the plants supplemented with HSLF. HSLF supplementation at 3.0 g L^−1^ in the non–salt-treated plants was associated with higher K^+^ content in the roots and leaves. Although the Na^+^ and K^+^ contents significantly varied across treatments, the K^+^/Na^+^ ratios of the roots and leaves of the non-salt-treated plants did not vary significantly with the HSLF concentration. However, this ratio was significantly smaller in the salt-stressed plants than in the control plants. By contrast, regardless of the dose, a significantly lower increase in this ratio was observed in the leaves of the HSLF-treated plants. 

### 3.3. Between-Group Differences in Cell Morphology of Lettuce Root Apices

**Figure 1:**
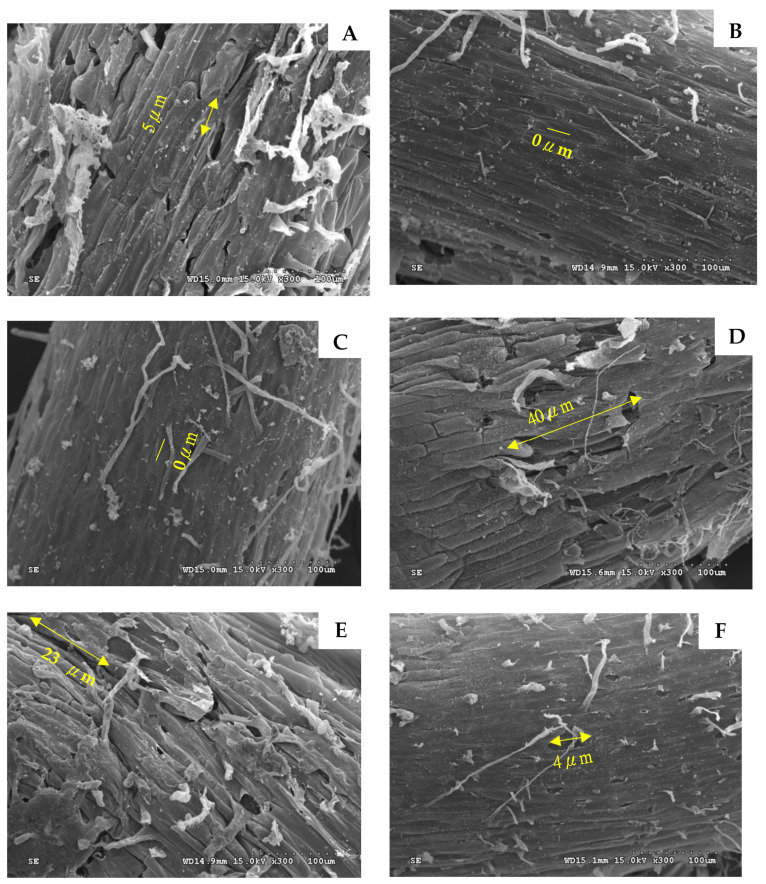
SEM images of lettuce root apices with different salt and sulfur fertilizer treatments. (**A**) S_0_H_0_, (**B**) S_0_H_1.5_, (**C**) S_0_H_3.0_, (**D**) S_40_H_0_, (**E**) S_40_H_1.5_, (**F**) S_40_H_3.0_. S_0_ and S_40_ represent 0 and 40 mM NaCl, respectively. H_0_, H_1.5_, and H_3.0_ represent 0, 1.5, and 3.0 g L^−1^ HSLF fertilizer, respectively.

The cells of the root apices remained normal and stable when not treated with salt (Figure 1A). The structure and arrangement of cells retained were preserved under different HSLF concentrations (Figure 1B,C). Conversely, the cells of the root apices shrank and disintegrated after 40 mM salt treatment. The high salt concentration caused dehydration of the cells (Figure 1D). However, when 1.5 g L^−1^ HSLF was added, the cells shrank only slightly (Figure 1E). With the HSLF treatment at 3.0 g L^−1^, the cells of the root apices of the lettuce repaired the salt injury and regained their integrity (Figure 1F).

## 4. Discussion

Supplementing saline soils with sulfur-containing compounds can prevent salt injury to plants because sulfur aids in the key mechanisms regulating the physiological processes that resist salt stress [18]. The present study’s plant morphology results suggest that supplementing soils with sulfur fertilizer minimizes the growth impairment usually associated with salinity. In the physiological response of plants to salinity, photosynthesis could be a means of resistance to salt stress [19]. Our results demonstrate that sulfur fertilizer supplementation can alleviate salt stress in lettuce cultivation. Similar results have been observed in a different lettuce cultivar [11] and in mustard plants [9]. Sulfur fertilizer may increase chlorophyll and soluble protein in lettuce [20], thereby establishing a balance between soluble protein and ribulose-1, 5-bisphosphate carboxylase/oxygenase in plant cells to resist salt injury [21]. In many plants, salinity may reduce nitrogen content, which is involved in Na^+^/NH_4_^+^ and Cl^−^/NO_3_^−^ antagonism [22,23]. Moreover, phosphorus content is lower in saline soils; however, sulfur supplementation can reverse this effect. Phosphorus is a key macronutrient in plants and is pivotal to their energy metabolism [24,25]. Because of the increase in Na^+^ and decrease in K^+^ content, the K^+^/Na^+^ ratio decreased significantly in our salt-stressed plants. However, the plants grown with a combination of salt and sulfur exhibited a considerably lower increase in this ratio (Table 2) because the sulfur supplement partially reversed the Na^+^ accumulation and K^+^ reduction caused by the salinity. Therefore, the results indicate that sulfur supplementation of lettuce plants decreases the uptake of toxic elements, such as Na^+^. In the treatment of 40 mM NaCl, Ca^2^^+^ and Mg^2+^ contents increased 8% and 7% more in the 1.5 g/L HSLF treatment than in non-HSLF and increased 11% and 10% more in the 3.0 g/L HSLF treatment than in non-HSLF (data not shown). This may indicate that sulfur as SO_4_^2−^ influenced the absorption and Na^+^ and increased the absorption of other cations. The promotion of K^+^ homeostasis by sulfur fertilizers in lettuce plants to resist salt stress is a noteworthy finding. Our findings suggest that 3.0 g L^−1^ sulfur supplementation is beneficial for lettuce plants grown in saline conditions. We contend that the appropriate absorption and usage of sulfur can achieve optimal lettuce plant growth and ionic homeostasis as well as maximize biomass production. Our results are in accord with those reported by Raza et al. (2018) for sesame plants [26]. Because sulfur fertilizer application accelerates plant growth and biomass production, it may benefit the availability and utilization of essential nutrients such as phosphorus and potassium (macronutrients that increased with sulfur fertilizer supplementation in lettuce). Such an increase may be due to sulfur metabolites resulting from sulfur supplementation and play a key role in plants under stressful conditions. Sulfur is an essential element of amino acids (i.e., cysteine and methionine), antioxidants (i.e., glutathione), and thioredoxin systems [27]. Additional use sulfur in plants can increase thiol content and protect barley plants from plant diseases [28]. According to our research, plants with higher levels of thiol compounds (e.g., sulfur-sufficient plants) are better equipped to resist toxic effects of salinity, a phenomenon our results may demonstrate. Plants develop oxidative mechanisms that protect them from salt injury, with the main antioxidants being glutathione and ascorbate [11].

SEM is an effective method for examining the morphology of plant cells [29]. In the present study, the morphology of lettuce root apices with different treatments was observed using SEM. Untreated by salt, the cells of the lettuce samples’ root apices retained greater structural integrity as the concentration of sulfur in the applied fertilizer increased. The cell cracking was as small as 5 μm in the treatments of S_0_F_0_ to 0 μm in S_0_F_1.5_ and S_0_F_3.0_ (Figure 1A–C). However, the cell cracking became larger in the S_40_F_0_ treatment (40 μm) than in S_40_F_1.5_ (20 μm) and in S_40_F_3.0_ (4 μm) (Figure 1D–F). It showed that the integrity of cells gradually recovered after an increase in the supplement of sulfur fertilizer. In general, the integrity of cells is related to the sulfur content of metabolites (cysteine, glutathione, S-adenosylmethionine), and the depletion of glutathione affects the redox and stress response systems of the glutathione–ascorbate cycle. Zhao et al. (2014) asserted that sulfur can promote cell elongation related to a substantial increase in cell-wall-bound hydroxyproline [30]. Sulfur might release auxin-like plant growth promoters and enhance biochemical activity in maize [31], suggesting that some signaling pathways activated by sulfur are expressed at a posttranscriptional level through calcium-dependent protein phosphorylation. These effects are mediated by hormonal signaling pathways and dominated by an upregulation of genes responsive to the synthesis of auxin in corn and tomatoes and of other genes that encode metabolic pathways for the uptake of nutrients, consequently alleviating salt stress [32].

## 5. Conclusions

Sulfur fertilizer supplementation attenuated the deleterious effects of salinity on the hydroponic growth of lettuce plants. Compounds containing sulfur from hoggeries should be used for fertilizer and biomaterial. The effect of sulfur may be related to a high K^+^/Na^+^ ratio and enhanced phosphorus and potassium uptake. SEM analysis indicated that the apices of lettuce roots were injured by salt treatment, and sulfur could reduce such injury.

## Figures and Tables

**Table 1:** Plant height, root length, dry weight, and leaf nutrition data of lettuce treated with two salt concentrations and three sulfur fertilizer concentrations.

Treatment	Plant Height (cm)	Root Length (cm)	FW ^1^ (g)	N (%)	P (%)	K (%)
S_0_H_0_	7.9 ± 0.7 a ^2^	18.1 ± 0.8 ab	51.4 ± 3.1 ab	3.92 ± 0.4 a	0.47 ± 0.02 a	2.99 ± 0.5 a
S_0_H_1.5_	8.3 ± 0.8 a	18.7 ± 0.7 a	58.2 ± 3.5 a	3.85 ± 0.3 a	0.49 ± 0.03 a	3.15 ± 0.4 a
S_0_H_3.0_	8.1 ± 0.6 a	18.7 ± 0.9 a	62.8 ± 4.1 a	3.77 ± 0.3 a	0.52 ± 0.03 a	3.39 ± 0.4 a
S_40_H_0_	7.1 ± 0.5 b	17.2 ± 0.5 b	43.6 ± 3.0 b	2.83 ± 0.4 b	0.32 ± 0.04 b	2.59 ± 0.3 b
S_40_H_1.5_	7.3 ± 0.5 b	17.6 ± 0.6 ab	47.8 ± 4.2 b	2.91 ± 0.3 b	0.34 ± 0.03 b	2.99 ± 0.4 a
S_40_H_3.0_	7.8 ± 0.3 a	18.0 ± 0.4 ab	49.5 ± 3.7 ab	3.12 ± 0.2 ab	0.38 ± 0.02 b	3.16 ± 0.5 a

^1^ FW: fresh weight. ^2^ Different letters indicate significantly different results by LSD tests at *p* < 0.05. S_0_ and S_40_ represent 0 and 40 mM NaCl, respectively; H_0_, H_1.5_, and H_3.0_ represent 0, 1.5, and 3.0 g L^−1^ HSLF, respectively.

**Table 2:** Influence of salt stress and sulfur supplementation on sodium, potassium, and potassium/sodium ratio in lettuce roots and leaves.

Treatments	Root			Leaf		
	K (mg/g DW ^1^)	Na (mg/g DW)	K/Na	K (mg/g DW)	Na (mg/g DW)	K/Na
S_0_H_0_	27.85 ± 0.58 a ^2^	5.88 ± 0.14 b	4.73 ± 0.15 a	29.91 ± 0.34 ab	4.71 ± 0.11 b	6.35 ± 0.24 b
S_0_H_1.5_	27.93 ± 0.35 a	5.93 ± 0.16 b	4.71 ± 0.13 a	31.46 ± 0.22 ab	4.80 ± 0.13 c	6.55 ± 0.21 ab
S_0_H_3.0_	28.17 ± 0.56 a	6.06 ± 0.18 b	4.65 ± 0.17 a	33.88 ± 0.18 a	4.76 ± 0.04 c	7.12 ± 0.09 a
S_40_H_0_	19.76 ± 0.43 b	12.05 ± 0.42 a	1.64 ± 0.08 c	25.94 ± 0.53 b	16.11 ± 0.29 a	1.61 ± 0.03 d
S_40_H_1.5_	23.08 ± 0.94 ab	11.74 ± 0.15 a	1.97 ± 0.09 c	29.92 ± 0.07 ab	15.23 ± 0.35 a	1.96 ± 0.05 c
S_40_H_3.0_	24.41 ± 0.29 ab	10.12 ± 0.08 a	2.41 ± 0.11 b	31.60 ± 0.11 ab	14.85 ± 0.30 ab	2.12 ± 0.06 c

^1^ DW: dry weight ^2^ Different letters indicate significantly different results by LSD tests at *p* < 0.05. S_0_ and S_40_ represent 0 and 40 mM NaCl, respectively; H_0_, H_1.5_, and H_3.0_ represent 0, 1.5, and 3.0 g L^−1^ HSLF, respectively.

## Data Availability

Data is contained within the article.
